# A cross-sectional mental-health survey of Chinese postgraduate students majoring in stomatology post COVID-19 restrictions

**DOI:** 10.3389/fpubh.2024.1376540

**Published:** 2024-05-03

**Authors:** Yuwei Zhang, Yue Jia, MaErWa MuLaTiHaJi, Yiying Mi, Yukun Mei, Tianxiang Sun, Haibo Shi, Yifei Zhang, Yikun Zhang, Rui Zou, Lin Niu, Shaojie Dong

**Affiliations:** ^1^Key Laboratory of Shaanxi Province for Craniofacial Precision Medicine Research, College of Stomatology, Xi’an Jiaotong University, Xi’an, China; ^2^Clinical Research Center of Shaanxi Province for Dental and Maxillofacial Diseases, Xi’an, China; ^3^Health Science Center, Xi’an Jiaotong University, Xi’an, China; ^4^College of Stomatology, China Medical University, Shenyang, China; ^5^Department of Prosthodontics, College of Stomatology, Xi’an Jiaotong University, Xi’an, China

**Keywords:** mental health, COVID-19 restrictions, postgraduate, stomatology, China

## Abstract

**Background:**

The psychological status of Chinese postgraduate students majoring in stomatology after the COVID-19 restrictions still remains unclear. The objective of this study is to evaluate the mental status through a cross-sectional survey and gather related theoretical evidence for psychological intervention on postgraduate students majoring in stomatology.

**Methods:**

An online survey was administered, and subjective well-being, anxiety, stress and depression symptoms were assessed using the 5-item World Health Organization Well-Being Index (WHO-5), item Generalized Anxiety Disorder Scale (GAD-7), 10-item Perceived Stress Scale (PSS-10), and Patient Health Questionnaire-9 (PHQ-9), respectively, wherein suicidal ideation and sleep-related problems were measured with PHQ-9 and Insomnia Severity Index (ISI).

**Results:**

A total of 208 participants who completed one questionnaire were considered as valid. It was found that female respondents generally exhibited significantly higher levels of PSS-10, PHQ-9, and GAD-7 scores and shorter physical activity hours than male students. Students from rural areas demonstrated significantly higher levels of PHQ-9, suicidal ideation, and less portion of good or fair family economic support. Additionally, individuals from only-child families reported increased levels of activity hours (1.78 ± 2.07, *p* = 0.045) and a higher portion (55.10%, *p* = 0.007) of having clear future plan as compared with multiple-child families. The risk factors for anxiety symptoms (GAD-7 score) were higher scores of PSS-10 (*OR* = 1.15, 95% CI = 1.09–1.22), PHQ-9 (*OR* = 1.35, 95% CI = 1.22–1.49), and ISI-7 (*OR* = 1.14, 95% CI = 1.06–1.23), while owning a clear graduation plan was the protective factor (*OR* = 0.55, 95% CI = 0.31–0.98). Moreover, the risk factors for depressive symptoms (PHQ-9) included PSS-10 (*OR* = 1.10, 95% CI = 1.04–1.16), GAD-7 (*OR* = 1.38, 95% CI = 1.25–1.52), suicidal ideation (*OR* = 5.66, 95% CI = 3.37–9.51), and ISI-7 (*OR* = 1.17, 95% CI = 1.09–1.25). Approximately 98.08% of Chinese postgraduates studying stomatology reported experiencing at least moderate stress after the COVID-19 restrictions.

**Conclusion:**

Within the limitations of this study, senior students were more inclined to stress, while anxiety symptoms were related to severer levels of stress, depression, and insomnia. Depressive symptoms were associated with higher levels of stress, anxiety, insomnia, suicidal ideation, and lower levels of self-reported well-being. Thus, psychological interventions for postgraduates should be timely and appropriately implemented by strengthening well-being, reasonably planning for the future, and good physique, thereby mitigating the psychological issues after COVID-19 restrictions.

## Introduction

In response to the outbreak of the coronavirus disease 2019 (COVID-19) pandemic, college students in China and globally encountered multiple instances all over the world had experienced several episodes of school closures. These measures were implemented as a part of general broader containment measures to slow strategies aimed at mitigating the spread of COVID-19 (1, 2). In an effort to mitigate the ongoing worldwide outbreak and protect college students from COVID-19, all educational institutions have been closed at the first step, followed by a consistent restriction on off-campus travel. Given the significant investment in educational support aimed at enhancing the quality of education, most of universities possess multiple distinct functional campuses. However, postgraduate students frequently need to commute between these campuses to complete their studies, particularly for their graduation projects. With the restriction of off-campus, most of students experienced their laboratories closed, projects disrupted, collaborations halted, and financial resources strained. Medical students, being a unique subset within this global pandemic, have to act dual roles as students and potential medical personnel. They had been demonstrated by several analyses that they are at a higher risk of developing mental problems during COVID-19 crisis (3–5). Given the unique nature of stomatology education, the mental issues of postgraduates majored in this subject could not be ignored, especially when the mandatory standardized training initiative for dentists was temporarily halted as a result of stomatology hospitals, either ceasing or partially curtailing their medical services during the COVID-19 pandemic.

It was widely recognized that the COVID-19 pandemic has led to a decrease in virulence and an increase in population immunity. To return to normalcy in both individual life and economy activities, China concluded its nationwide restriction measures on 20 December 2022 (6). However, it remains uncertain whether the impact of COVID-19 will diminish after the restrictions. Previous research studies revealed that there was a slight improvement in mental health since the end of restrictions while still at a lower level than that before the pandemic (7, 8). After the ending of COVID-19 restrictions, students are confronted with a shift in teaching modalities from online and remote learning to on-site instruction, which necessitates a swift adaptation as early in their academic careers when they were not impacted by the pandemic to make up for any missed courses and technical practices within a limited timeframe. Additionally, after the full non-restriction, the inevitable COVID-19 infection adds to the stresses of life events. Therefore, the mental status of Chinese postgraduates majoring in stomatology and how it could provide useful guidance in real world are worth investigating. Now it is the first year after the nationwide restrictions in China, when it is crucial to assess whether the mental state of oral postgraduates has returned to pre-pandemic levels. Furthermore, it is important to understand the potential long-term effects of COVID-19 on the academic journey of medical students.

Therefore, the primary objective of this study was to evaluate the mental health status of Chinese postgraduates majoring in stomatology in 2023, marking the first year abolishing the COVID-19 pandemic restrictions in China. In especial, we sought to investigate the prevalence of anxiety and depressive symptoms, perceived stress, and sleep-related issues. Furthermore, we aimed to analyze the corresponding measurements based on the potential confounding factors and their associations. This research represents a further step toward gaining a more profound understanding of the long-term implications of the COVID-19.

## Methods

### Samples

The present cross-sectional survey was conducted from 15 October 2023 to 30 December 2023, a year subsequent to the restriction imposed in late 2022 in China. Sample size was calculated according to the formula: $n=\frac{Z_{1-\frac{\alpha }{2}}^{2}\times p\times \left(1-p\right)}{d^{2}}$, wherein 5% α error probability was taken, and $Z_{1-\frac{\alpha }{2}}^{2}$ was 1.96; the expected incidence rate *p* was set as 0.67, given the prevalence rate of mental health problems among Chinese medical college students (9), and the results of our pilot study conducted in Xi’an, China, among postgraduate medical students during COVID-19; *d* (allowable error) was taken as 0.1*p.* A sample size of at least 189 respondents was needed.

Subsequently, 25 colleges/universities were selected randomly for this study via the random-number method by numbering nationwide colleges/universities that offering a stomatology postgraduate program. Then, stratified sampling was carried out. The selection process employed a convenience sampling method with a balanced distribution of participant numbers.

### Measurements

#### General demographic characteristics

General information such as current educational institutions, age, gender, grade, place of residence, household formation (multiple-child family or the only-child family), and self-assessed family economic status was recorded accordingly as basic background features.

#### Well-being

The universal and its Chinese version of the 5-item World Health Organization Well-Being (WHO-5) Index, comprising five self-rating items on a six-point scale, were utilized to assess the general well-being of included participants (10, 11). Each item is scaled from 0 (no time at all) to 5 (all the time) based on the sentiment experienced over the preceding 2 weeks. Subsequently, a cumulative score for all items was calculated ranging from 0 (absence of well-being) to 25 (maximum well-being).

#### Anxiety

General anxiety disorder and its severity were assessed using the universal and Chinese version of the Generalized Anxiety Disorder-7 (GAD-7). The GAD-7 is comprised of a combination of seven items rated on a four-point scale ranging from 0 (not at all) to 3 (nearly every day). The total score, ranging from 0 to 21 with a cutoff score of 11 points, was assessed for clinically relevant general anxiety disorder symptoms (8, 12).

#### Stress

The universal and its Chinese version of the 10-item Perceived Stress Scale (PSS-10) were utilized to assess the self-reported stress levels of the respondents. The PSS-10 comprises 10 items rated on a five-point Likert scale ranging from 1 (never) to 5 (always), reflecting the occurrence frequency of each item in the preceding month (13, 14). Scores for six negative items, along with four inverted positive items, were calculated, which lied between 0 (the lowest possible level) and 40 (the highest level of perceived stress). A cutoff score of 14 was established to define moderate stress (8).

#### Depression

The Patient Health Questionnaire-9 (PHQ-9) was employed to evaluate depressive symptoms. The PHQ-9 comprises nine items, based on the DSM-IV criteria for depression module ranging from 0 (not at all) to 3 (nearly every day), leading to a score from 0 to 27. A clinical threshold of 11 points is established as indicative of depressive symptoms in adolescents (8, 15, 16).

#### Suicidal ideation

Suicidal ideation was assessed using the ninth item of the PHQ-9: “*How often have you been bothered by thoughts that you would be better off dead or of rather hurting yourself in some way over the last two weeks?”* A two-point score was set as a cutoff for severe suicidal ideations (8).

#### Insomnia

The nature, severity, and impact of sleep-related problems were assessed using the Insomnia Severity Index (ISI). The ISI comprises four-point self-report having 7 items ranging from 0 to 28 with a cutoff point of 15 designated as moderate insomnia (8, 17).

#### Smartphone usage

Smartphone usage appears to be linked with mental health, and excessive use tends to be associated with negative consequences (i.e., technology-related addiction risk) as included in our measurements (18). The frequency of smartphone usage was divided into six options, namely, <1 h/day, 1–2 h/day, 3–4 h/day, 5–6 h/day, 7–8 h/day, and > 8 h/day (8).

#### Physical activity

Physical activity appears to be also related to mental health, which is often described as a protective factor. Thus, it was included in our measurements (19). The frequency of physical activity was self-reported from number 0 to 7, which represented the number of days with over 60 min of physical activity per week based on the WHO recommendations for healthy physical activity purpose (20).

#### Self-perceived source of pressure

Self-perceived Source of Pressure was represented by multiple choice questions based on our pilot study in the initial smaller scale population. Additionally, the options were summarized as “worried about academic scores”; “worried about the result of graduation project”; “worried about not being able to graduate”; “worried about not being able to find a job”; and “worried that one’s abilities may not meet the requirements of the employer after employment”.

#### Future plan

Future plan was assessed based on whether a definitive decision was made or not and then what the decision entailed, such as pursuing a full time/on-the-job PhD, working directly or not.

#### Measurement reliability

The internal consistency and reliability of the instruments/measurements were assessed by Cronbach’s alpha (α) coefficient.

#### Statistical analysis

All the continuous data were displayed as mean ± standard deviation (SD) and median, while categorical data were reported as number and its percentage. T-tests and non-parametric Mann–Whitney U rank-sum tests were utilized for mean comparison between two groups depending on normality (Kolmogorov–Smirnov normality test when *n* > 50) and homogeneity of variance (Levene’s test). Comparisons among multiple groups were evaluated by one-way analysis of variance (ANOVA), if the statistical indicators satisfied the normality. The statistical significances between groups were compared with the Fisher post-hoc least significant difference test (LSD, when equal-variance assumed) or Dunnett’s T3 test (when equal-variance not assumed). Otherwise, Kruskal–Wallis H rank sum test was selected for comparison between multiple independent samples. R × C table chi-square tests were conducted to compare the prevalence of the related psychological symptoms across groups. Multivariate logistic regression models were utilized for the association between various factors and stress/depression/anxiety. Variance inflation factor (VIF) analysis was conducted before applying multivariate logistic regression models so as to guarantee that no multicollinearity was found. Statistical analyses were conducted by SPSS 22.0 (SPSS Inc., Chicago, IL, USA). *p* < 0.05 (two-tailed) represented statistically significant.

### Data collection and ethical approval

The study was approved by the Medical Ethical Committee of Stomatology Hospital of Xi’an Jiaotong University, China (No. 191), and was conducted in accordance with the Declarations of Helsinki. The online Questionnaire Star was utilized via an anonymous, self-rated method.^1^ All the participants were given assurance of confidentiality that the information gathered would be used exclusively for research purposes. Electronic informed consent was obtained from all participants prior to our data collection. To eliminate repeated respondents, each device was limited to one response. Logic check was proceeded by two independent reviewers to avoid invalid questionnaires. Only valid questionnaires were used for further analysis. The online questionnaires encompassed general background demographic features and assessed the impact of COVID-19 restriction on life, particularly the related psychological symptoms and sources of respondents. The aforementioned items of each measurement revealed in the content of questionnaires are shown in Supplementary Table S1.

## Results

Finally, 208 out of 235 questionnaires were identified as valid ones for further analysis. Based on Cronbach’s α coefficient results, the measurements utilized in the sample presented good internal consistency and reliability (Supplementary Table S2). The sample of oral medicine postgraduate respondents consisted of 208 individuals, with a gender distribution of 138 women (66.35%) and 70 men (33.65%). When categorizing the data based on gender, a significant higher level of self-reported stress (f−m: 30.19 ± 7.85–26.29 ± 6.80, *p* < 0.0001), depression (f−m: 7.61 ± 5.62–6.06 ± 5.50, *p* = 0.044), and generalized anxiety disorder (f−m: 76.45 ± 5.22–4.63 ± 4.51, *p* = 0.017) were found in women. Meanwhile, we observed a significant decreased level of physical time per week (f−m: 1.01 ± 1.34–2.26 ± 2.23, *p* < 0.0001) in female students compared with male students. Additionally, most of the women had clear future plan to get a job (74.58%) after graduation, while only 48.57% of men chose to work and 45.71% of men decided to pursue a PhD degree (*χ*^2^ = 6.652, *p* = 0.036). When considering the place of residence, an increased level of PHQ-9 (depressive symptoms, R−U: 8.26 ± 5.61–6.40 ± 5.52, *p* = 0.013) and suicidal ideation (PHQ-No.9 score, R−U: 0.45 ± 0.77–0.23 ± 0.52, *p* = 0.014) in students from rural areas than that from urban areas was observed. Furthermore, the basic structure of self-perceived family economic status (*χ*^2^ = 24.933, *p* < 0.0001) and the proportion of students with a clear graduation plan (*χ*^2^ = 3.847, *p* = 0.050) differed in these two areas. A significant higher ratio of students had a clear plan when they are in the famlies of fair/good economic status or from urban areas. Intriguingly, when data from respondents were divided according to their household formation, a longer physical time per week (one-multiple: 1.78 ± 2.07–1.12 ± 1.43, *p* = 0.045) was observed in the only-child families after the restrictions. Students from the only-child families appeared to be more inclined to make clear further plan (*p* = 0.007). Furthermore, it was noticeable that the total average of PSS-10 scores and the individual score of 98.08% of the participants exceeded the cutoff point, indicating a wide range of participants experienced at least moderate stress (the total and individual analyses are presented in Table 1).

**Table 1:** Outcomes of the psychological variables based on gender, place of residence, and household formation.

		Gender				Place of residence				Household formation			
	Total	Female	Male	Statistics	*p* value	Rural	Urban	Statistics	*p* value	The only child	Multiple-child family	Statistics	*p* value
*N*	208	138	70			77	131			98	110		
WHO-5 score													
mean ± SD (median)	12.54 ± 5.51 (12.00)	12.38 ± 5.48 (11.50)	12.86 ± 5.61 (13.00)	*D* = 0.083 *F* = 0.038	0.584	11.97 ± 5.55 (12.00)	12.88 ± 5.49 (12.00)	*D* = 0.116 *Z* = −1.125	0.261	12.50 ± 5.70 (12.00)	12.58 ± 5.36 (12.00)	*D* = 0.062 *F* = 0.019	0.915
PSS-10 score													
mean ± SD (median)	28.88 ± 7.72 (29.00)	30.19 ± 7.85 (30.00)	26.29 ± 6.80 (27.00)	*D* = 0.052 *F* = 2.238	<0.0001***	29.78 ± 7.92 (30.00)	28.34 ± 7.58 (28.00)	*D* = 0.078 *F* = 0.452	0.196	28.66 ± 7.49 (29.00)	29.06 ± 7.95 (29.00)	*D* = 0.082 *F* = 0.502	0.710
≥14, No. (%)	204 (98.08)	137 (99.28)	67 (95.71)	*χ*^2^ = 1.520	0.218	76 (98.70)	128 (97.71)	*χ*^2^ = 0.0001	1.000	96 (97.96)	109 (99.09)	*χ*^2^ = 0.010	0.920
PHQ-9 score													
mean ± SD (median)	7.09 ± 5.62 (6.00)	7.61 ± 5.62 (7.00)	6.06 ± 5.50 (4.00)	*D* = 0.099 *Z* = −2.014	0.044*	8.26 ± 5.61 (8.00)	6.40 ± 5.52 (5.00)	*D* = 0.092 *Z* = −2.480	0.013*	6.51 ± 5.63 (5.00)	7.60 ± 5.58 (7.00)	*D* = 0.147 *Z* = −1.522	0.128
≥11, No. (%)	55 (26.44)	38 (27.54)	17 (24.29)	*χ*^2^ = 0.252	0.615	22 (28.57)	33 (25.19)	*χ*^2^ = 0.285	0.593	28 (28.57)	27 (24.55)	*χ*^2^ = 0.432	0.511
Suicidal ideation (PHQ-No.9 score)													
mean ± SD (median)	0.31 ± 0.64 (0.00)	0.28 ± 0.60 (0.00)	0.37 ± 0.71 (0.00)	*D* = 0.463 *Z* = −0.764	0.445	0.45 ± 0.77 (0.00)	0.23 ± 0.52 (0.00)	*D* = 0.490 *Z* = −2.452	0.014*	0.29 ± 0.63 (0.00)	0.34 ± 0.65 (0.00)	*D* = 0.442 *Z* = −0.777	0.437
≥2, No. (%)	14 (6.73)	7 (5.07)	7 (10.00)	*χ*^2^ = 1.097	0.295	7 (9.09)	7 (5.34)	*χ*^2^ = 1.085	0.298	7 (7.14)	7 (6.36)	*χ*^2^ = 0.050	0.823
GAD-7 score													
mean ± SD (median)	5.84 ± 5.06 (6.00)	6.45 ± 5.22 (7.00)	4.63 ± 4.51 (4.50)	*D* = 0.152 *Z* = −2.394	0.017*	6.44 ± 4.99 (7.00)	5.48 ± 5.08 (6.00)	*D* = 0.161 *Z* = −1.677	0.094	5.34 ± 4.80 (5.00)	6.28 ± 5.26 (7.00)	*D* = 0.173 *Z* = −1.397	0.162
≥11, No. (%)	30 (14.42)	22 (15.94)	8 (11.43)	*χ*^2^ = 0.767	0.381	13 (16.88)	17 (12.98)	*χ*^2^ = 0.599	0.439	13 (13.27)	17 (15.45)	*χ*^2^ = 0.201	0.654
ISI-7 score													
mean ± SD (median)	6.38 ± 5.15 (6.00)	6.55 ± 5.25 (6.00)	6.04 ± 4.97 (5.00)	*D* = 0.119 *Z* = −0.701	0.483	6.99 ± 5.70 (6.00)	6.02 ± 4.79 (6.00)	*D* = 0.167 *Z* = −1.015	0.310	6.17 ± 4.97 (5.50)	6.56 ± 5.33 (6.00)	*D* = 0.136 *Z* = −0.485	0.628
≥15, No. (%)	20 (9.62)	15 (10.87)	5 (7.14)	*χ*^2^ = 0.742	0.389	3 (3.90)	2 (1.53)	*χ*^2^ = 0.370	0.543	9 (9.18)	11 (10.00)	*χ*^2^ = 0.040	0.842
Smartphone usage													
No. (%)	<1 h/day	<1 h/day	<1 h/day	χ ^2^ = 18.62	0.002*	<1 h/day	<1 h/day	χ ^2^ = 7.700	0.174	<1 h/day	<1 h/day	χ^2^ = 13.648	0.018*
	14 (6.73)	3 (2.17)	11 (15.71)			2 (2.60)	12 (9.16)			13 (13.27)	1 (0.91)		
	1–2 h/day	1–2 h/day	1–2 h/day			1–2 h/day	1–2 h/day			1–2 h/day	1–2 h/day		
	19 (9.13)	10 (7.25)	9 (12.86)			10 (12.99)	9 (6.87)			9 (9.18)	10 (9.09)		
	3–4 h/day	3–4 h/day	3–4 h/day			3–4 h/day	3–4 h/day			3–4 h/day	3–4 h/day		
	59 (28.37)	44 (31.88)	15 (21.43)			25 (32.47)	34 (25.95)			26 (26.53)	33 (30.00)		
	5–6 h/day	5–6 h/day	5–6 h/day			5–6 h/day	5–6 h/day			5–6 h/day	5–6 h/day		
	61 (29.33)	45 (32.61)	16 (22.86)			18 (23.38)	43 (32.82)			27 (27.55)	34 (30.91)		
	7–8 h/day	7–8 h/day	7–8 h/day			7–8 h/day	7–8 h/day			7–8 h/day	7–8 h/day		
	27 (12.98)	20 (14.49)	7 (10.00)			10 (12.99)	17 (12.98)			13 (13.27)	14 (12.73)		
	>8 h/day	>8 h/day	>8 h/day			>8 h/day	>8 h/day			>8 h/day	>8 h/day		
	28 (13.46)	16 (11.59)	12 (17.14)			12 (15.58)	16 (12.21)			10 (10.20)	18 (16.36)		
Physical activity (days more than 60 min per week)													
mean ± SD (median)	1.42 ± 1.79 (1.00)	1.01 ± 1.34 (0.00)	2.26 ± 2.23 (2.00)	*D* = 0.288 *Z* = −4.218	<0.0001 ***	1.04 ± 1.35 (1.00)	1.66 ± 1.97 (1.00)	*D* = 0.246 *Z* = −1.79	0.074	1.78 ± 2.07 (1.00)	1.12 ± 1.43 (1.00)	*D* = 0.217 *Z* = −2.003	0.045*
Self-perceived family economic status													
No. (%)	good	good	good	*χ*^2^ = 0.694	0.707	good	good	*χ*^2^ = 24.933	<0.0001 ***	good	good	*χ*^2^ = 3.426	0.180
	17 (8.17)	11 (7.97)	6 (8.57)			3 (3.90)	14 (10.69)			11 (11.22)	6 (5.45)		
	fair	fair	fair			fair	fair			fair	fair		
	161 (77.40)	109 (78.99)	52 (74.29)			51 (66.23)	110 (83.97)			76 (77.55)	85 (77.27)		
	bad	bad	bad			bad	bad			bad	bad		
	30 (14.42)	18 (13.04)	12 (17.14)			23 (29.87)	7 (5.34)			11 (11.23)	19 (17.27)		
Having clear graduation plan													
No. (%)	Yes	yes	yes	*χ*^2^ = 0.985	0.321	yes	yes	*χ*^2^ = 3.847	0.050*	yes	yes	*χ*^2^ = 7.347	0.007**
	94 (45.19)	59 (42.75)	35 (50.00)			28 (36.36)	66 (50.38)			54 (55.10)	40 (36.36)		
	*No*	*No*	*No*			*No*	*No*			*No*	*No*		
	114 (54.81)	79 (57.25)	35 (50.00)			49 (63.64)	65 (49.62)			44 (44.90)	70 (63.64)		
Graduation plan													
No. (%)	PhD	PhD	PhD	*χ*^2^ = 6.652	0.036*	PhD	PhD	*χ*^2^ = 4.714	0.095	PhD	PhD	*χ*^2^ = 3.062	0.216
	30 (31.91)	14 (23.73)	16 (45.71)			6 (21.43)	24 (36.36)			21 (38.89)	9 (22.50)		
	Work	Work	Work			*Work*	*Work*			Work	Work		
	61 (64.89)	44 (74.58)	17 (48.57)			22 (78.57)	39 (59.09)			30 (55.56)	29 (72.50)		
	Others	Others	Others			Others	Others			*Others*	*Others*		
	3 (3.19)	1 (1.69)	2 (5.72)			0 (0.00)	3 (4.55)			3 (5.56)	2 (5.00)		
Current sources of pressure													
No.	No job 138	No job 98	No job 40	*χ*^2^ = 2.231	0.693	No job 55	No job 83	*χ*^2^ = 0.327	0.988	No job 58	No job 80	χ^2^ = 1.202	0.878
	Requirements of the employer 134	Requirements of the employer 95	Requirements of the employer 39			Requirements of the employer 52	Requirements of the employer 82			Requirements of the employer 57	Requirements of the employer 77		
	Unable to graduate 103	Unable to graduate 77	Unable to graduate 26			Unable to graduate 42	Unable to graduate 61			Unable to graduate 43	Unable to graduate 60		
	Scores 55	Scores 37	Scores 18			Scores 23	Scores 32			Scores 19	Scores 36		
	Graduation Project 95	Graduation Project 73	Graduation Project 22			Graduation Project 36	Graduation Project 59			Graduation Project 38	Graduation Project 57		

M, mean score; SD, standard deviation; D, test statistics of normality based on Kolmogorov–Smirnov test (when *n* > 50). F, test statistics of Levene’s test for equality of variances. Z, test statistics of Mann–Whitney rank-sum tests. *χ*^2^, Chi-square test statistics. WHO-5, World Health Organization Wellbeing Index; PHQ-9, Patient Health Questionnaire 2 Scale; GAD-7, General Anxiety Disorder 7 Scale; ISI, Insomnia Severity Index Score; PSS-10, Perceived Stress Scale 10; PhD, Doctor of Philosophy. Suicidal ideations: item 9 of the PHQ-9: “Thoughts that you would be better off dead or of hurting yourself.” *p < 0.05, **p < 0.01, and ***p < 0.001.

Grade was another essential variable in our study. The number of students in Grade1, Grade 2, and Grade 3 was 61, 65, and 82, respectively (Table 2). The age of students from each grade was significantly different (*p* < 0.0001). In general, the scores of PSS-10 and GAD-7 showed a hierarchic ascending trend from Grade 1 to 3, with significantly higher levels of stress and anxiety symptoms in Grade 3 than in Grade 1 (P1-3 < 0.05). The same trend was found in the level of PHQ-9, with significantly more severe depressive symptoms in Grade 3 and 2 than in Grade 1 (P1-2 = 0.027, P1-3 = 0.007). Students in Grade 3 (54.88%) were more likely to have a clear further plan (χ^2^ = 10.183, *p* = 0.006).

**Table 2:** Outcomes of the psychological variables based on grade.

	Grade 1	Grade 2	Grade 3	Statistics	*p* value
*N*	61	65	82		
Age	24.39 ± 1.74 (24.00)	24.35 ± 2.20 (24.00)	25.27 ± 1.85 (25.00)	*Z* = 16.58	<0.0001***
WHO-5	12.43 ± 5.26 (13.00)	12.60 ± 5.17 (11.00)	12.61 ± 6.00 (12.50)	F′ = 0.02 F1-2 = 0.08 F1-3 = 1.43 F2-3 = 2.19	P1-2 = 0.878 P1-3 = 0.849 P2-3 = 0.966
PSS-10					
mean ± SD (median)	27.57 ± 6.49 (28.00)	28.71 ± 8.02 (29.00)	29.98 ± 8.23 (30.00)	F′ = 1.73 F1-2 = 1.59 F1-3 = 5.52 F2-3 = 0.74	P1-2 = 0.386 P1-3 = 0.050* P2-3 = 0.350
≥14, No. (%)	61 (100.00)	63 (96.92)	80 (97.56)	χ^2^ = 2.87	0.239
PHQ-9					
mean ± SD (median)	5.43 ± 5.20 (4.00)	7.55 ± 5.65 (7.00)	7.95 ± 5.68 (8.00)	Z1-2 = −2.21 Z1-3 = −2.70 Z2-3 = −0.51	P1-2 = 0.027* P1-3 = 0.007* P2-3 = 0.608
≥11, No. (%)	13 (21.31)	16 (24.62)	26 (31.71)	χ^2^ = 2.11	0.349
Suicidal ideation (PHQ-No.9 score)					
mean ± SD (median)	0.21 ± 0.49 (0.00)	0.31 ± 0.61 (0.00)	0.39 ± 0.75 (0.00)	Z1-2 = −0.78 Z1-3 = −1.33 Z2-3 = −0.54	P1-2 = 0.436 P1-3 = 0.183 P2-3 = 0.589
≥2, No. (%)	2 (3.28)	5 (7.69)	7 (8.54)	*χ*^2^ = 1.90	0.387
GAD-7 score					
mean ± SD (median)	4.98 ± 4.96 (5.00)	5.77 ± 4.69 (6.00)	6.52 ± 5.35 (7.00)	Z1-2 = −1.26 Z1-3 = −2.03 Z2-3 = −1.00	P1-2 = 0.207 P1-3 = 0.043* P2-3 = 0.316
≥11, No. (%)	5 (8.20)	7 (10.77)	18 (21.95)	*χ*^2^ = 6.38	0.041*
ISI-7 score					
mean ± SD (median)	5.57 ± 5.61 (4.00)	6.63 ± 4.66 (6.00)	6.78 ± 5.17 (6.50)	Z1-2 = −1.82 Z1-3 = −1.82 Z2-3 = −0.04	P1-2 = 0.069 P1-3 = 0.068 P2-3 = 0.967
≥15, No. (%)	8 (13.11)	5 (7.69)	7 (8.54)	*χ*^2^ = 1.25	0.536
Smartphone usage					
No. (%)	<1 h/day	<1 h/day	<1 h/day	*χ*^2^ = 46.95	<0.0001***
	13 (21.31)	0 (00.00)	1 (1.22)		
	1–2 h/day	1–2 h/day	1–2 h/day		
	4 (6.56)	1 (1.54)	14 (17.07)		
	3–4 h/day	3–4 h/day	3–4 h/day		
	13 (21.31)	21 (32.31)	25 (30.49)		
	5–6 h/day	5–6 h/day	5–6 h/day		
	13 (21.31)	24 (36.92)	24 (29.27)		
	7–8 h/day	7–8 h/day	7–8 h/day		
	10 (16.39)	6 (9.23)	11 (13.41)		
	>8 h/day	>8 h/day	>8 h/day		
	8 (13.11)	13 (20.00)	7 (8.54)		
Physical activity (days more than 60 min per week)					
mean ± SD (median)	1.69 ± 2.33 (1.00)	1.31 ± 1.56 (1.00)	1.33 ± 1.47 (1.00)	Z1-2 = −0.05 Z1-3 = −0.10 Z2-3 = −0.23	P1-2 = 0.959 P1-3 = 0.920 P2-3 = 0.818
Self-perceived family economic status					
No. (%)	good	good	good	*χ*^2^ = 3.43	0.489
	3 (4.92)	4 (6.15)	10 (12.20)		
	fair	fair	fair		
	50 (81.97)	52 (80.00)	59 (71.95)		
Having clear graduation plan					
No. (%)	Yes	Yes	Yes	*χ*^2^ = 10.18	0.006**
	30 (49.18)	19 (29.23)	45 (54.88)		
	No	No	No		
	31 (50.82)	46 (70.77)	37 (45.12)		
Graduation plan					
No. (%)	PhD	PhD	PhD	*χ*^2^ = 7.50	0.112
	14 (46.67)	4 (21.05)	11 (24.44)		
	Work	Work	Work		
	14 (46.67)	13 (68.42)	33 (73.33)		
	Others	Others	Others		
	2 (6.67)	2 (10.53)	1 (2.22)		
Current sources of pressure					
No.	No job 33	No job 46	No job 59	*χ*^2^ = 7.53	0.480
	Requirements of the employer 42	Requirements of the employer 42	Requirements of the employer 50		
	Unable to graduate 25	Unable to graduate 30	Unable to graduate 48		
	Scores 12	Scores 17	Scores 26		
	Graduation Project 20	Graduation Project 39	Graduation Project 36		

Data are expressed as the mean ± standard deviation (median). F, test statistics of ANOVA. F, test statistics of Levene’s test for equality of variances. Z, test statistics of Mann–Whitney rank-sum tests. *χ*^2^, Chi-square test statistics. P_n1−n2_: *p*-value based on LSD or Dunnett or Mann–Whitney rank-sum tests between groups of n1 and n2. WHO-5, World Health Organization Well-being Index; PHQ-9, Patient Health Questionnaire 2 Scale; GAD-7, General Anxiety Disorder 7 Scale; ISI, Insomnia Severity Index Score; PSS-10, Perceived Stress Scale 10; PhD, Doctor of Philosophy. Suicidal ideations: item 9 of the PHQ-9: “Thoughts that you would be better off dead or of hurting yourself.” *p < 0.05, **p < 0.01, and ***p < 0.001.

VIF analysis was conducted before applying multivariate logistic regression models, and no multicollinearity was found (Supplementary Tables S3–S5). Subsequently, considering the factors influencing college students’ stress (PSS-10 score, Table 3), a multivariate logistic regression analysis was conducted. Compared with younger students, senior/older students exhibited a higher risk of self-reported stress symptoms (*OR* = 1.15, 95% *CI*: 1.01–1.30), wherein age was a significant variable rather than grade level. Furthermore, the longer hours of physical activity per day (*OR* = 0.73, 95% *CI*: 0.63–0.84) and having a clear graduation plan (*OR* = 0.49, 95% *CI*: 0.29–0.81) were associated with lower possibility of stress symptoms.

**Table 3:** Multivariate logistic regression of factors influencing college students’ stress (PSS-10 score).

Variables			*β*	Std. error	Wald	OR	95%CI
Age			0.14	0.07	4.47	1.15*	1.01, 1.30
Grade	Grade 1	Reference					
	Grade 2		0.04	0.32	0.01	1.04	0.56, 1.94
	Grade 3		0.37	0.31	1.46	1.45	0.79, 2.65
Gender	Male	Reference					
	Female		0.35	0.28	1.59	1.42	0.82, 2.46
Self-perceived family economic status	Bad	Reference					
	Fair		0.45	0.35	1.63	1.57	0.79, 3.11
	Good		−0.28	0.54	9.26	0.76	0.26, 2.19
Physical activity days			−0.32	0.08	17.91	0.73***	0.63, 0.84
Smartphone usage hours			0.03	0.09	0.08	0.96	0.79, 1.16
Having clear graduation plan	No	Reference					
	Yes		−0.72	0.26	7.57	0.49**	0.29, 0.81

*p < 0.05, **p < 0.01, ***p < 0.001.

Moreover, students with higher level of PSS-10 score (*OR* = 1.15, 95% *CI*: 1.09–1.22), PHQ-9 score (*OR* = 1.35, 95% *CI*: 1.22–1.49), and ISI-7 score (*OR* = 1.14, 95% *CI*: 1.06–1.23) presented a higher risk of anxiety. Meanwhile, students with clear graduation plan would suffer from less anxiety (*OR* = 0.55, 95% *CI*: 0.31–0.98) (Table 4).

**Table 4:** Multivariate logistic regression of factors influencing college students’ anxiety (GAD-7 score).

Variables			*β*	Std. error	Wald	OR	95%CI
Age			−0.10	0.07	1.76	0.91	0.79, 1.05
Grade	Grade 1	Reference					
	Grade 2		−0.59	0.35	2.80	0.56	0.28, 1.11
	Grade 3		0.03	0.34	0.01	1.03	0.53, 2.02
Gender	Male	Reference					
	Female		0.11	0.31	0.14	1.12	0.61, 2.05
Self-perceived family economic status	Bad	Reference					
	Fair		0.68	0.39	3.03	1.97	0.92, 4.23
	Good		0.26	0.62	0.18	1.30	0.38, 4.42
WHO-5 score			−0.05	0.03	2.97	0.95	0.89, 1.01
PSS-10 score			0.14	0.03	25.08	1.15***	1.09, 1.22
PHQ-9 score			0.30	0.05	32.59	1.35***	1.22, 1.49
Suicidal ideation (PHQ-No.9 score)			0.13	0.28	0.21	1.14	0.66, 1.96
ISI-7 score			0.13	0.04	12.49	1.14***	1.06, 1.23
Physical activity days			0.02	0.09	0.03	1.02	0.86, 1.20
Smartphone usage hours			0.02	0.10	0.04	1.02	0.83, 1.25
Having clear graduation plan	No	Reference					
	Yes		−0.60	0.30	4.12	0.55*	0.31, 0.98

*p < 0.05, ***p < 0.001.

In addition, those who reported a higher level of PSS-10 (*OR* = 1.10, 95% *CI*: 1.04–1.16), GAD-7 (*OR* = 1.38, 95% *CI*: 1.25–1.52), suicidal ideation (*OR* = 5.66, 95% *CI*: 3.37–9.51), and ISI-7 (*OR* = 1.17, 95% *CI*: 1.09–1.25), which could be regarded as risk factors, were associated with a higher risk of depression compared with those with lower levels. Moreover, higher levels of WHO-5 scores were related to lower degree of depression (*OR* = 0.94, 95% *CI*: 0.89–0.99), as shown in Table 5.

**Table 5:** Multivariate logistic regression of factors influencing college students’ depression (PHQ-9 score).

Variables			*β*	Std. error	Wald	OR	95%CI
Age			−0.04	0.07	0.40	0.96	0.84, 1.09
Gender	Male	Reference					
	Female		0.13	0.29	0.19	1.14	0.64, 2.01
Grade	Grade 1	Reference					
	Grade 2		0.51	0.33	2.40	1.67	0.87, 3.19
	Grade 3		0.58	0.32	3.23	1.79	0.95, 3.37
Self-perceived family economic status	Bad	Reference					
	Fair		−0.59	0.37	2.50	0.56	0.27, 1.15
	Good		−0.61	0.57	1.16	0.54	0.18, 1.66
WHO-5 score			−0.06	0.03	3.98	0.94*	0.89, 0.99
PSS-10 score			0.09	0.03	11.33	1.10***	1.04, 1.16
GAD-7 score			0.32	0.05	40.97	1.38***	1.25, 1.52
Suicidal Ideation (PHQ-No.9 score)			1.73	0.27	42.73	5.66***	3.37, 9.51
ISI-7 score			0.16	0.03	20.16	1.17***	1.09, 1.25
Physical activity days			−0.10	0.08	1.47	0.91	0.78, 1.06
Smartphone Usage hours			0.16	0.10	2.68	1.18	0.97, 1.43
Having Clear Graduation Plan	No	Reference					
	Yes		−0.37	0.28	1.76	0.67	0.40, 1.20

*p < 0.05, ***p < 0.001.

## Discussion

In this research, the sample of oral medicine postgraduate respondents consisted of 208 individuals, with a distribution of 66.35% as women and 33.65% as men. This distribution closely aligns with enrollment gender ratio of the annual master in China.

During the pandemic, all educational institutions suspended classes or constantly restricted off-campus travel to mitigate the nationwide and global spread of the virus and protect college students from COVID-19. Given the significant investment in educational support to enhance educational quality, most universities operate multiple functional campuses. Postgraduate students often need to commute between these campuses to complete their studies, particularly for their graduation projects. With the restriction on off-campus activities, most students faced challenges such as closed laboratories, obstructed projects, suspended cooperation, and financial strain on families. Due to the unique nature of the oral medicine postgraduate training program, the mandatory standardized training project for dentists was halted as stomatology hospitals either suspended or partially resumed their medical services, considering that the aerogel in the clinics would accelerate the spread of virus during the COVID-19 pandemic. The impact of the pandemic on professional skills and academic performance was significant, indicating that prolonged isolation, restricted movement within confined spaces, and online remote learning have negative emotional implications (21, 22).

Considering weakened virulence and enhanced immunity in the population of COVID-19, nationwide non-restriction was implemented to restore normal citizen life and the national economy (6). However, whether the effect of COVID-19 ever vanished after the restrictions on 20 December 2022 in China still remains unclear. As approaching the first anniversary for nationwide non-restriction, our study found that 14.42% of postgraduate students reported clinical general anxiety, which was significantly lower than the investigation results derived from Chinese college students (41.1%) during the epidemic (23). It is reasonable to surmise that the outbreak of COVID-19 indicated the peak value of incidence and prevalence of anxiety, and there was a noticeable decline following the period of normalization with unrestricted social activity. However, the after effect of COVID-19 is inevitable and should be taken seriously.

The transformation of teaching mode from online learning and remote schooling to on-site learning required students to make up for the missed courses and technical practices in a short time, together with the inevitable COVID-19 infection after the full non-restriction, serving as the sources of stressful life events. Thus, we found approximately 98.08% of Chinese postgraduates majoring in stomatology experienced at least moderate stress after the COVID-19 restrictions. Consistent with previous studies (8, 24), women in our studies were more likely to suffer from stress, depression and anxiety, and less active in physical exercise. Female postgraduate students often face challenges, such as marital pressure, age-appropriate childbirth, pressure from the family of origin, and differential treatment in the workplace. Therefore, these factors could potentially influence gender differences in the relationship between life stressors and the onset of psychosis. Additionally, these variables along with the confounding factor of age in the female group might also explain why our real-world results indicate that most of the women having a clear future plan tended to work (74.58%) after graduation and 48.57% of men chose to work, while 45.71% of men decided to pursue a PhD degree (*χ*^2^ = 6.652, *p* = 0.036).

Considering the subjective well-being and stressful life events among rural/urban Chinese postgraduates, it was observed that students from rural areas tended to be more depressive, and their suicidal ideation was worth noticeable. The economic status disparity has mentioned in these two regions, and our included population was consistent with the traditional economic pattern of Chinese rural and urban areas. The basic higher ratio of structure of fair/good self-perceived family economic status was found in urban (*χ*^2^ = 24.933, *p* < 0.0001) areas. Students from rural areas are more prone to depression unlikely to make a determined graduation plan, and their suicidal ideation was worth noticeable in case these of stressful life events, leading to more depression and less life satisfaction among Chinese rural-to-urban migrant college students. This may attribute to the gap in urban–rural economic development (25).

Intriguingly, when data from respondents were categorized based on their household formation, longer physical time per week (one-multiple: 1.78 ± 2.07–1.12 ± 1.43, *p* = 0.045) was observed in the only-child families after the restrictions. Students from these only-child families seemed to be more inclined to make a clear future plan (*p* = 0.007). It could be attributed to the more family support in traditional one-child Chinese family structure, where all the paternal care and attention were focused on the only child. Consequently, more resource allocation and assistance during growth events can facilitate early goal and ideal setting.

Multivariate logistic regression analysis was a valid tool for fully utilizing the information of data, and eliminating/controlling the influence of confounding variables with an obtained adjusted OR, thereby explaining the relationship between the response variable and the influencing factors after adjusting for confounding factors. Therefore, logistic regression model was selected for assessing the factors that influence the aimed mental symptoms and utilize influencing factors to facilitate the establishment of subsequent symptom prediction models. Our study revealed that senior students were more prone to stress before January, a period when neither normal job nor study have been settled. Stress symptoms were associated with greater age, less physical activity days, and undetermined future plan, while anxiety symptoms were inclined with greater stress, depression, insomnia level, and undetermined future plan. Depressive symptoms were also associated with greater stress, anxiety, insomnia level, suicidal ideation, and lower level of self-reported well-being.

Generally, high resilience was negatively correlated with suicidal ideation, while academic stress was not negatively correlated only when participants with low or moderate levels of resilience showed a positive association between stress and suicidal ideation (26). However, approximately 98.08% of Chinese postgraduates studying stomatology experienced at least moderate stress after the COVID-19 restrictions, most of whom were considered as having high resilience through regular psychological assessment. However, it is important to notice that suicidal ideation could be heightened by increased academic stress with associated anxiety and depression among postgraduates. Thus, it is necessary to timely guide them, culturing with ideal adaptive coping strategies and greater resilience to mitigate the tendency of academic stress leading to suicidal ideation.

In summary, the present study exhibited several strengths. First, based on the enrollment scale and distribution of dental majors’ postgraduates in China, it represented the most extensive and proportional sample survey on the mental health of dental postgraduates after the COVID-19 pandemic restrictions. Second, the study reveals a significant proportion of mental issues among postgraduate students. Inspired by the findings of this study, we can make valuable suggestions for psychological interventions on postgraduates, which should be timely and appropriately implemented by strengthening well-being and good physique to reduce the psychological harm after the COVID-19 restrictions, when students bothered by the epidemic start to live up with its aftereffect. And provided related guidance for preventing postgraduate students’ mental problems, especially for these key susceptible groups and at some essential timepoints, including medical students, female groups, students in Grade 3 or from rural areas and timepoint before graduation earlier than January when job and current/further study were neither settled.

However, the sample size of this study was limited, and the sampling number of 25 universities was limited as well. Considering the aim of this study, the selected universities merely included those undergraduate institutions providing a postgraduate program in stomatology, without vocational and technical colleges. The career plans and psychological qualities of graduates from different universities vary greatly. In addition, given that this is a cross-sectional study, the actual cause-effect could not be inferred. Although logistic regression models were utilized in this study for better control of confounding factors, some mediating variables cannot be fully explained. All these findings should only be interpreted with caution due to potential confounding factors and temporal variations. More rigorous follow-up and intervention research are needed for cause-effect and temporal variation analysis.

## Conclusion

Approximately 98.08% of Chinese postgraduates studying stomatology reported experiencing at least moderate stress, following COVID-19 restrictions. Female students were found more likely to suffer from stress, depression and anxiety, and less energetic during physical exercise. Students from rural areas tended to be more depressive, and their suicidal ideation was worth noticeable. Senior students were particularly prone to stress before January when neither employment nor studies had been secured. Risk factors for stress symptoms included older age and higher levels of depression and anxiety. Meanwhile, risk factors for anxiety symptoms included higher levels of stress, depression, and insomnia. Protective factors for reliving anxiety included owning clear graduation plan timely. Depressive symptoms were associated with higher levels of stress, anxiety, insomnia, and suicidal ideation and lower levels of subjective well-being. Therefore, timely and appropriate psychological interventions for postgraduates should implemented by strengthening subjective well-being and good physique to reduce the psychological harm after the COVID-19 restrictions as students begin to cope with the aftermath of the epidemic.

## Data availability statement

The original contributions presented in the study are included in the article/Supplementary material, further inquiries can be directed to the corresponding authors.

## Ethics statement

The studies involving humans were approved by Medical Ethical Committee of Stomatology Hospital of Xi’an Jiaotong University. The studies were conducted in accordance with the local legislation and institutional requirements. The participants provided their written informed consent to participate in this study.

## Author contributions

YuZ: Writing – original draft, Methodology, Data curation, Conceptualization. YJ: Writing – original draft, Methodology, Data curation, Conceptualization. MM: Writing – original draft, Methodology, Data curation, Conceptualization. YMi: Writing – review & editing, Investigation, Data curation. YMe: Writing – review & editing, Investigation, Data curation. TS: Writing – review & editing, Investigation, Data curation. HS: Writing – review & editing, Investigation, Data curation. YifZ: Writing – review & editing, Investigation, Data curation. YikZ: Writing – review & editing, Investigation, Data curation. RZ: Writing – review & editing, Supervision, Methodology. LN: Writing – review & editing, Funding acquisition. SD: Writing – review & editing, Writing – original draft, Funding acquisition.

## Footnotes

^1^ https://www.wjx.cn
