# Supplementary material for: A cross-sectional mental-health survey of Chinese postgraduate students majoring in stomatology post COVID-19 restrictions
Source: Front Public Health. 2024 May 3;12:1376540. doi: 10.3389/fpubh.2024.1376540 (PMC11099282; doi:10.3389/fpubh.2024.1376540)
Supplement: Supplementary file 1 [file Table_1.docx]

**A Cross-sectional Mental-health Survey of Chinese Postgraduate Students Majoring in Stomatology Post COVID-19 Restrictions**

Table S1 The content of questionnaires

| No. | Content |
| --- | --- |
| 1 | Current school |
| 2 | Gender (Male/Female) |
| 3 | Age |
| 4 | Grade (1/2/3) |
| 5 | Place of residence (Urban/Rural) |
| 6 | Household formation (The only child/Multiple-child family) |
| 7 | Family economic status (Good/fair/bad) |
| 8 | Over the past 2 weeks…  1 ... I have felt cheerful and in good spirits  2 ... I have felt calm and relaxed  3 ... I have felt active and vigorous  4 ... I woke up feeling fresh and rested  5 ... my daily life has been fi lled with things that interest me  (5 All of the time/4 Most of the time/3 More than half the time/2 Less than half the time/1 Some of the time/0 At no time) |
| 9 | Over the last 2 weeks, how often have you been bothered by the following problems?  1. Feeling nervous, anxious or on edge  2. Not being able to stop or control worrying  3. Worrying too much about different things  4. Trouble relaxing  5. Being so restless that it is hard to sit still  6. Becoming easily annoyed or irritable  7. Feeling afraid as if something awful might happen  (0 Not at all/1 Several days/2 More than half the days/3 Nearly every Day) |
| 10 | Over the past 1 month…  1. Been upset  2. Unable to control  3. Stressed  4. Felt confident  5. Going your way  6. Could not cope  7. Control irritations  8. On top of things  9. Been angered  10. Could not overcome  (1 never/2 occasionally/3 sometimes/4 often/5 always)  The ten items included six negative items (1, 2, 3, 6, 9, and 10), interpreted as perceived helplessness, and four positive items (4, 5, 7, and 8), interpreted as perceived selfefficacy. The scores for positive items need to be inverted to calculate the total score and higher scores indicate higher levels of perceived stress. |
| 11 | Over the past 2 weeks…  1.Little interest or pleasure in doing things  2. Feeling down, depressed, or hopeless  3. Trouble falling or staying asleep, or sleeping too much  4. Feeling tired or having little energy  5. Poor appetite or overeating  6. Feeling bad about yourself, or that you are a failure or have let yourself or your family down  7. Trouble concentrating on things, such as watching television  8. Moving or speaking so slowly that other people could have noticed or being so fidgety or restless that you have been moving around a lot more than usual  9. Thoughts that you would be better off dead or of hurting yourself in some way  (0 not at all/1 Several days/2 More than half the days/ 3 nearly every day) |
| 12 | Over the past 1 month…  1. falling asleep  2. staying asleep  3. early awakening  4. satisfaction  5. interference  6. noticeable  7. worry  *Items 1-3 0, no problem; 1, mild; 2, moderate; 3, severe; 4, very severe  *Item 4  0, very satisfied; 1, satisfied; 2, neutral; 3, dissatisfied;  4, very dissatisfied  *Items 5-7 0, not at all; 1, a little; 2, somewhat; 3, much; 4, very much |
| 13 | Smartphone usage  The frequency of smartphone usage was divided into: <1 h/day, 1–2 h/day, 3–4 h/day, 5–6 h/day, 7–8 h/day, and >8 h/day |
| 14 | Physical activity  The frequency of physical activity was self-reported from number 0 - 7, which represented the number of days with over 60 minutes of physical activity per week |
| 15 | Do you currently have a clear plan for the future?  (1 Yes/2 No)  If *Yes*, what is your future plan?  (1 pursuing a full time or on-the-job PhD/2 working directly/3 neither) |
| 16 | What is your main source of pressure?  (1 Worried about academic scores/2 Worry about the result of graduation project/3 Worried about not being able to graduate/4 Worried about not being able to find a job/5 Worried that one’s abilities may not meet the requirements of the employer after employment) |

Table S2. Internal consistency and reliability assessed by Cronbach’s α coefficient in each measurement

| Measurement | α |
| --- | --- |
| WHO-5 | 0.885 |
| GAD-7 | 0.903 |
| PSS-10 | 0.832 |
| PHQ-9 | 0.738 |
| ISI | 0.920 |

WHO-5: World Health Organization Wellbeing Index, GAD-7: General Anxiety Disorder 7 Scale, PSS-10: Perceived Stress Scale 10, PHQ-9: Patient Health Questionnaire 2 Scale, ISI: Insomnia Severity Index Score.

Table S3. Collinearity Statistics (Dependent Variable: PSS-10 score)

| Variables | | | Tolerance | VIF |
| --- | --- | --- | --- | --- |
| Age |  |  | 0.92 | 1.09 |
| Grade | Grade 1 | Reference |  |  |
|  | Grade 2 |  | 0.73 | 1.37 |
|  | Grade 3 |  | 0.74 | 1.35 |
| Gender | Male | Reference |  |  |
|  | Female |  | 0.84 | 1.20 |
| Self-perceived family economic status | Bad | Reference |  |  |
|  | Fair |  | 0.96 | 1.05 |
|  | Good |  | 0.96 | 1.04 |
| Physical activity days |  |  | 0.86 | 1.17 |
| Smartphone Usage hours |  |  | 0.87 | 1.15 |
| Having Clear Graduation Plan | No | Reference |  |  |
|  | Yes |  | 0.87 | 1.14 |

VIF: Variance Inflation Factor.

Table S4. Collinearity Statistics (Dependent Variable: GAD-7 score)

| Variables | | | Tolerance | | VIF |
| --- | --- | --- | --- | --- | --- |
| Age |  |  | 0.89 | | 1.13 |
| Grade | Grade 1 | Reference |  |  |  |
|  | Grade 2 |  | 0.72 | | 1.40 |
|  | Grade 3 |  | 0.70 | | 1.43 |
| Gender | Male | Reference |  |  |  |
|  | Female |  | 0.81 | | 1.24 |
| Self-perceived family economic status | Bad | Reference |  |  |  |
|  | Fair |  | 0.93 | | 1.08 |
|  | Good |  | 0.91 | | 1.10 |
| WHO-5 score |  |  | 0.60 | | 1.66 |
| PSS-10 score |  |  | 0.39 | | 2.58 |
| PHQ-9 score |  |  | 0.22 | | 4.58 |
| Suicidal Ideation (PHQ-No.9 score) |  |  | 0.50 | | 2.02 |
| ISI-7 score |  |  | 0.46 | | 2.16 |
| Physical activity days |  |  | 0.78 | | 1.29 |
| Smartphone Usage hours |  |  | 0.83 | | 1.20 |
| Having Clear Graduation Plan | No | Reference |  |  |  |
|  | Yes |  | 0.78 | | 1.29 |

VIF: Variance Inflation Factor.

Table S5. Collinearity Statistics (Dependent Variable: PHQ-9 score)

| Variables | | | Tolerance | VIF |
| --- | --- | --- | --- | --- |
| Age |  |  | 0.89 | 1.13 |
| Gender | Male | Reference |  |  |
|  | Female |  | 0.81 | 1.24 |
| Grade | Grade 1 | Reference |  |  |
|  | Grade 2 |  | 0.71 | 1.41 |
|  | Grade 3 |  | 0.72 | 1.40 |
| Self-perceived family economic status | Bad | Reference |  |  |
|  | Fair |  | 0.93 | 1.08 |
|  | Good |  | 0.90 | 1.12 |
| WHO-5 score |  |  | 0.61 | 1.64 |
| PSS-10 score |  |  | 0.36 | 2.78 |
| GAD-7 score |  |  | 0.29 | 3.41 |
| Suicidal Ideation (PHQ-No.9 score) |  |  | 0.65 | 1.54 |
| ISI-7 score |  |  | 0.50 | 2.00 |
| Physical activity days |  |  | 0.78 | 1.29 |
| Smartphone Usage hours |  |  | 0.84 | 1.19 |
| Having Clear Graduation Plan | No | Reference |  |  |
|  | Yes |  | 0.77 | 1.29 |

VIF: Variance Inflation Factor.
